# Proteomic profiling of protein expression changes after 3 months-exercise in ESRD patients on hemodialysis

**DOI:** 10.1186/s12882-023-03146-w

**Published:** 2023-04-21

**Authors:** Hye Yun Jeong, Hyun-Ju An, Min Ji Sung, Min Heui Ha, Yu Ho Lee, Dong Ho Yang, Tae Young Yang, Dohyun Han, So-Young Lee

**Affiliations:** 1 Division of Nephrology, Department of Internal Medicine, CHA University School of Medicine, CHA Bundang Medical Center, 59 Yatap-ro, Bundang-gu, Seongnam-si, 13496 Republic of Korea; 2 Transdisciplinary Department of Medicine & Advanced Technology, Seoul National University Hospital, 101 Daehak-ro, Seoul, 03080 Republic of Korea; 3 Proteomics Core Facility, Biomedical Research Institute, Seoul National University Hospital, Seoul, Republic of Korea

**Keywords:** Chronic kidney disease, hemodialysis, Proteomics, End-stage renal disease

## Abstract

The prevalence of chronic kidney disease (CKD) is steadily increasing, and it is a global health burden. Exercise has been suggested to improve physical activity and the quality of life in patients with CKD, eventually reducing mortality. This study investigated the change in physical performance after exercise in dialysis-dependent patients with CKD and analyzed differentially expressed proteins before and after the exercise. Plasma samples were collected at enrollment and after 3 months of exercise. Liquid chromatography with tandem mass spectrometry analysis and data-independent acquisition results were analyzed to determine the significantly regulated proteins. A total of 37 patients on dialysis were recruited, and 16 were randomized to exercise for 3 months. The hand grip strength and the walking speed significantly improved in the exercise group. Proteome analysis revealed 60 significantly expressed proteins after 3 months of exercise. In the protein functional analysis, the significantly expressed proteins were involved in the immune response. Also, some of the key significantly expressed proteins [(M Matrix metallopeptidase 9 (MMP-9), Activin A Receptor Type 1B (ACVR1B), Fetuin B (FETUB)] were validated via an enzyme-linked immunosorbent assay. Our results showed that exercise in dialysis-dependent patients with CKD could improve their physical performance. These results indicated that this beneficial effect of exercise in these populations could be associated with immune response.

**Supplementary Information:**

The online version contains supplementary material available at 10.1186/s12882-023-03146-w.

## Introduction

The global prevalence of chronic kidney disease (CKD) has increased, and it is considered the leading cause of public health problems [1]. The increase in the incidence of this disease affects the global burden of mortality and morbidity, the main cause of which is cardiovascular disease (CVD).

Recent studies have shown that high physical function and activity are associated with improved survival and decreased mortality in patients with CKD [2, 3]. These studies emphasize the importance of exercise interventions for improving physical function and activity levels in these patients. Exercise interventions improve aerobic capacity, walking capacity, muscular function, and health-related quality of life, resulting in reduced cardiovascular risk and mortality rates in the studied population [4]. In a pilot study involving patients with CKD, aerobic exercise training improved the peak oxygen uptake, physical impairment, and arterial stiffness, decreasing cardiovascular and mortality risk [5]. A systemic review also showed that regular exercise training in patients with CKD is related to improved health outcomes. Although the beneficial effects of exercise in patients with CKD have been reported, studies on the molecular response to exercise interventions in these populations are scarce [6].

Therefore, this study investigated the change in physical performance after exercise intervention in patients with CKD on dialysis. We also analyzed protein expression after exercise intervention using a proteomic approach.

## Materials and methods

### Study population

**Fig. 1:**
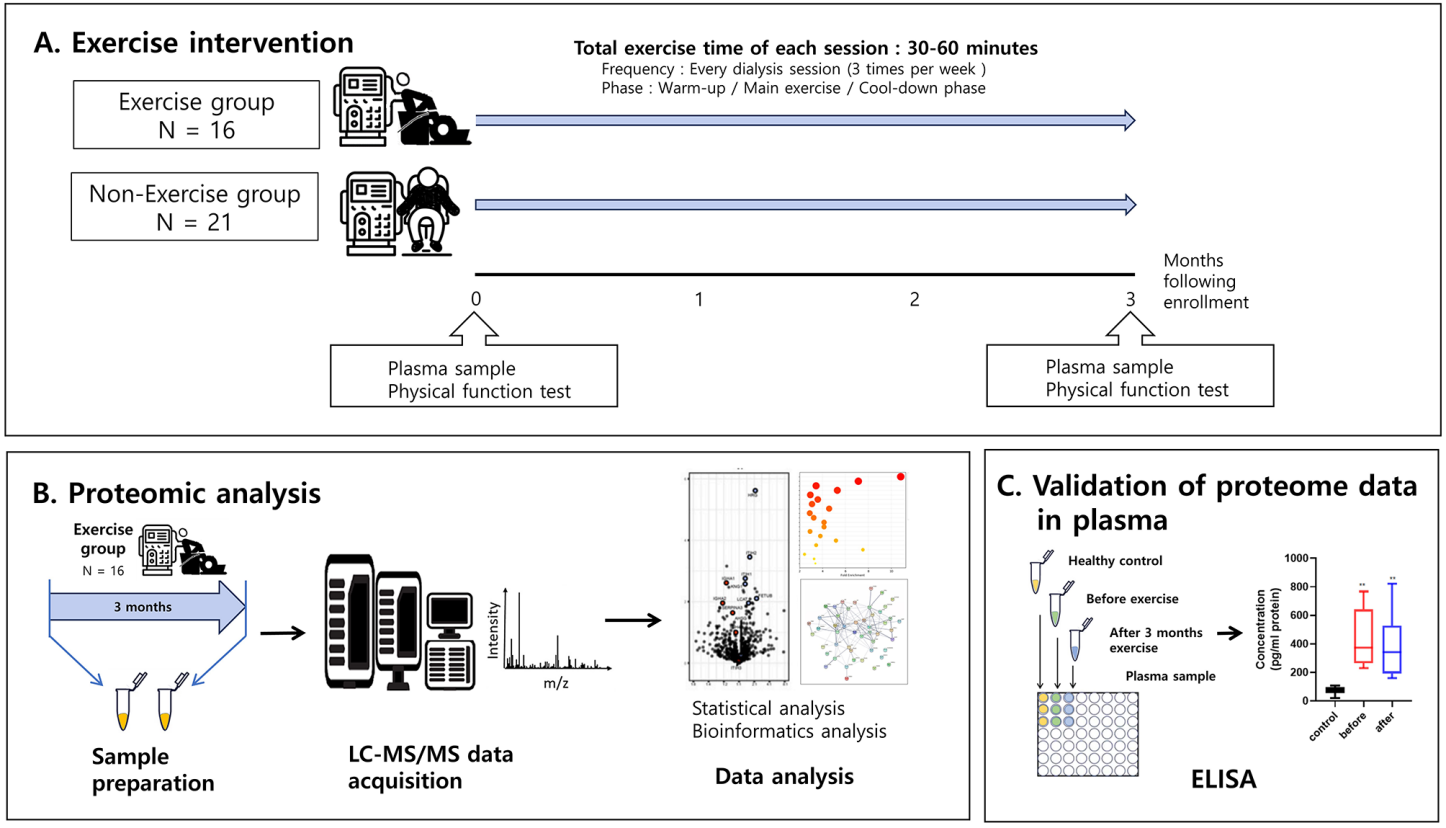
Schematic diagram summarizing the study design. (**A**) Total 37 patients were recruited and 16 were randomized for 3 months exercise during each hemodialysis session. (**B**) The plasma samples for proteomic analysis were collected at the enrollment time and 3 months later in exercise group. The LC-MS/MS analysis methods was performed and the DIA data from individual samples were analyzed to determine significantly regulated protein. The bioinformatics analysis was used for protein functional annotation and protein–protein interaction. (**C**) To validate proteome data, the ELISA analysis was used for plasma sample of exercise group. The change of protein expression levels was also compare to the sample from age-matched healthy controls

This study prospectively enrolled ESRD patients in CHA bundang medical center in South Korea, and undergoing maintenance hemodialysis for at least 3 months was diagnostic criteria for ESRD. The overall scheme of this study is shown in Fig. 1. In September 2016, this study prospectively enrolled patients with end-stage renal disease (ESRD) treated with maintenance hemodialysis at the CHA Bundang Medical Center, South Korea. The inclusion criteria were adult patients older than 18 years and undergoing hemodialysis three times per week (> 12 h/week) for at least three months. Patients with a history of active infection, coagulation disorders, cancer, or kidney transplantation were excluded from the study.

Initially, 37 adult patients were recruited, and 16 were randomized for three months of exercise during hemodialysis sessions (Fig. 1). The plasma samples for proteomic analysis were collected at the enrollment time and after three months exercise. To validate the protein expression levels, plasma samples were additionally collected from age-matched healthy controls, who underwent health examinations at CHA Bundang Medical Center between January and February 2021.

This study was approved by the Institutional Review Board of CHA Bundang Medical Center and was conducted in accordance with the Declaration of Helsinki and principles of Good Clinical Practice (CHAMC 2016-05-064-024). Written informed consent was obtained from all the patients.

### Exercise intervention

The intradialytic exercise program consisted of aerobic exercises using a cycle ergometer. Each exercise phase consisted of a warm-up, main exercise, and cool-down phase. The warm-up consisted of stretches recommended by LORAC (2000), and was conducted for 5 min before the main exercise, sequencing from the upper body to lower body movements. In the main exercise phase, the patients were trained on a mechanically braked cycle ergometer (Mbike; Hong Jin Company, China), which was positioned in front of the dialysis recliner. The exercise was performed three times a week for every dialysis session and in the first 1–2 h of hemodialysis, with a total exercise time of 30–60 min. Using Borg’s 15-point scale for rating of perceived exertion, the patients were trained at a range of 7–9 for 5 min, range 12–15 for 20–50 min, and range 7–9 for 5 min. The cool-down phase was conducted in a manner similar to the warm-up phase for 5 min.

The training was terminated if the patient’s blood pressure exceeded (above 230 mmHg systolic blood pressure or above 120 mmHg diastolic blood pressure) or if the patient experienced dizziness, chest pain, nausea, vomiting, leg cramps, or severe dyspnea.

### Physical performance and body composition

The physical performance test was performed at the time of enrollment and three months later in both patient groups. Handgrip strength was measured immediately before the dialysis session. Patients performed three tests of maximum hand grip strength with the hand without vascular access using a Jamar hand dynamometer (Sammons Preston Inc., Bolingbrook, IL, USA). Slow walking speed was assessed by measuring gait speed over a 4 m course [7] [8]. Bioimpedance analysis (Inbody 620, In-body, Seoul, South Korea) was used to assess skeletal muscle mass, with measurement frequencies of 5, 50, and 500 kHz.

### Clinical variables

Patient demographics and clinical data, including age, sex, body mass index, and comorbidities, were obtained from medical records. CVD was defined as a medical history of congestive heart failure, angina pectoris, myocardial infarction, percutaneous transluminal coronary angioplasty, or coronary artery bypass surgery. Laboratory data were collected on hemoglobin, albumin, calcium, phosphorus, and creatinine levels at the time of patient enrolment and three months later in both patient groups.

### Sample preparation for proteomic analysis

To remove high-abundance proteins, 30 µL of plasma samples were diluted 1:4 with multiple affinity removal system (MARS) buffer A (Agilent Technologies, Santa Clara, CA, USA) and filtered with 0.22 μm Spin-X filters (Corning Costar, NY, USA). Individual plasma samples were depleted of six high-abundance human plasma proteins [albumin, Immunoglobulin (Ig) G, IgA, transferrin, haptoglobin, and antitrypsin) using a MARS column (Hu-6HC, 4.6 × 100 mm, Agilent Technologies, Santa Clara, CA, USA) on an Agilent 1260 HPLC system. Depleted plasma samples were concentrated by centrifugal filtration using a 3 kDa Amicon filter (Millipore, Burlington, MA, USA). Protein concentration was measured using the Bicinchoninic acid (BCA) assay.

For protein digestion, 100 µg of each sample was precipitated by adding a 5-fold volume of ice-cold acetone prior to digestion. The dried samples were reconstituted in 50 µL of SDT buffer (2% sodium dodecyl sulphate, 0.1 M dithiothreitol in 0.1 M Tris HCl pH 8.0). The denatured proteins were heated at 95 °C, and subsequently digested using a filter-aided sample preparation (FASP) method, as previously described [9], with some modifications. Briefly, protein samples were loaded onto a 30 K amicon filter (Millipore, Billerica, MA, USA), and the buffer was exchanged with UA solution (8 M urea in 0.1 M Tris-HCl pH 8.5) *via* centrifugation. After three buffer exchanges with UA solution, the reduced cysteines were alkylated with 0.05 M iodoacetamide in UA solution for 30 min in the dark at room temperature. Thereafter, the UA buffer was exchanged twice with 40 mM ammonium bicarbonate (ABC). Protein samples were digested with trypsin/LysC (enzyme to substrate ratio of 1:100) at 37 °C for 16 h. The resulting peptides were collected in new Eppendorf tubes *via* centrifugation, and an additional elution step was performed using 40 mM ABC and 0.5 M NaCl. All resulting peptides were acidified with 10% trifluoroacetic acid and desalted using in-house C18-StageTips, as described previously [10]. The desalted peptides were completely dried in a vacuum dryer and stored at − 80 °C.

### Liquid chromatography with tandem mass spectrometry analysis

Liquid Chromatography with tandem mass spectrometry (LC-MS/MS) analysis was performed using quadrupole Orbitrap mass spectrometers, Q-exactive plus (Thermo Fisher Scientific, Waltham, MA, USA), coupled to an Ultimate 3000 RSLC system (Dionex, Sunnyvale, CA, USA) with a nano-electrospray source as previously described, with some modifications [11]. Peptide samples were separated on a two-column setup with a trap column (300 μm I.D. × 5 mm, C18 3 μm, 100 Å) and an analytical column (75 μm I.D. × 50 cm, C18 1.9 μm, 100 Å). Prior to sample injection, the dried peptide samples were re-dissolved in solvent A (2% acetonitrile and 0.1% formic acid). After the samples were loaded onto the nano LC, a 90-min gradient from 8 to 30% solvent B (100% acetonitrile and 0.1% formic acid) was applied to all samples. The spray voltage was 2.0 kV in positive ion mode, and the temperature of the heated capillary was set to 320 °C. The hyper reaction monitoring (HRM) data-independent acquisition (DIA) method consisted of a survey scan at 35,000 resolution from 400 to 1,220 m/z (AGC target of 3 × 10^6^ or 60-ms injection time). Further, 19 DIA windows were acquired at a resolution of 35,000 with an automatic gain control target of 3e6 and auto injection time [11]. The stepped collision energy was 10% at 27%.

### Proteomic data processing

To generate spectral libraries, 24 data dependent acquisition (DDA) measurements were performed on the urine samples. DDA spectra were searched using MaxQuant against the UniProt Human Database (December 2014, 88,657 entries) and the indexed retention time standard peptide sequence. A spectral library was generated using the spectral library generation feature from Spectronaut 10 (Biognosys, Schlieren-Zurich, Switzerland) and DIA data from individual samples were analyzed. First, the DIA raw files were converted into HTRMS format using the GTRMS converter tool provided by Spectronaut. The false discovery rate (FDR) was estimated using the mProphe [12] approach and set to 1% at the peptide precursor and protein levels. The proteins were inferred by the software, and the quantification information was acquired at the protein level using a q-value < 0.01 criterion, which was used for the subsequent analyses.

### Statistical analyses of proteomics data

Statistical analyses of the DIA data were performed using Perseus software [13]. Initially, log_2_ transformation was conducted for these values because of the skewed data distribution. Valid values were filtered using proteins with a minimum of 50% quantified values in at least one group. Missing values were imputed based on a normal distribution (width = 0.3, downshift = 1.8) to simulate the signals of low-abundance proteins. Paired t-tests were performed for pairwise comparisons of proteomes to detect differentially expressed proteins (DEPs). Protein abundances were subjected to z-normalization, followed by hierarchical clustering using Pearson’s correlation distance.

### Bioinformatics analysis

Functional gene ontology (GO) and Kyoto Encyclopedia of Genes and Genomes (KEGG) pathway enrichment analyses of DEPs were performed using the DAVID bioinformatics tool (http://david.abcc.ncifcrif.gov/). Network analysis based on protein-protein interactions (PPI) was performed using the STRING database (http://string.embl.de/) to obtain additional information on the interaction network of DEPs. Significantly expressed proteins and DEPs were used, which had complex interactions.

### Enzyme linked immunosorbent assay (ELISA) for the validation of proteomic data

MMP-9 (cat. no. MBS2880173), ACVR1B (cat. no. MBS7232818), and FETUB (cat. no. MBS454400) concentrations in the plasma samples were measured according to the manufacturer’s specifications using MyBioSource ELISA kits. The minimum levels of detection were as follows: WFDC3, 3.12 ng/mL; FGFR1, 0.094 ng/mL; MMP-9, 0.056 ng/mL, ACVR1B, 0.5 ng/mL, FETUB, 0.04125 ng/mL.

### Statistical analysis

Categorical variables were recorded as numbers and percentages, and continuous variables were presented as mean ± standard variation or median. The χ^2^ test or Fisher’s exact test was used to compare the categorical variables. Continuous variables were compared using the Student’s t-test or Mann–Whitney *U* test. *P* value < 0.05 was considered statistically significant. Statistical analyses were performed using IBM SPSS Statistics for Windows version 21 (IBM Corp., Armonk, NY, USA).

## Results

### Baseline laboratory findings of included patients

Clinical characteristics and laboratory data are shown in Table 1. The mean age of the study participants was 57.4 ± 12.7 years, and 18% were male. The duration of dialysis was significantly longer in the non-exercise group (68.9 ± 47.9 vs. 28.1 ± 34.7 months, *p* = 0.005). The prevalence of hypertension (71.4 vs. 87.5%, *p* = 0.423) and diabetes mellitus (52.4 vs. 56.3%, *p* = 0.815) was higher in the exercise group than that in the non-exercise group, but the difference was not statistically significant. The hemoglobin (10.2 ± 1.0 vs. 10.8 ± 1.2 g/dL, *p* = 0.146) and serum albumin levels (3.8 ± 0.5 vs. 3.9 ± 0.2 g/dL, *p* = 0.477) were higher in the exercise group than that in the non-exercise group, but the differences were not statistically significant. The higher hand grip strength (26.1 ± 8.8 vs. 21.2 ± 7.1 kg, *p* = 0.076) in the non-exercise group and faster walking speed (1.31 ± 0.4 vs. 1.23 ± 0.29 m/s, *p* = 0.478) in the exercise group at baseline were not statistically significant (Table 1).

**Table 1:** Baseline characteristics of study participants

Characteristics	Overall	control	exercise	*P*-value
	(n = 37)	(n = 21)	(n = 16)	
Male, n (%)	18 (48.6)	10 (47.6)	8 (50.0)	0.886
Age, years	57.4 ± 12.7	56.8 ± 12.3	58.2 ± 13.5	0.740
HD duration, months	51.2 ± 46.9	68.9 ± 47.9	28.1 34.7	0.005
Blood flow rate (ml/min)	278.4 ± 23.0	276.2 ± 24.4	281.3 ± 21.6	0.516
Hypertention, n (%)	24 (64.9)	15 (71.4)	14 (87.5)	0.423
Diabetes, n (%)	25 (67.6)	11 (52.4)	9 (56.3)	0.815
Cerebrovascular diseases, n (%)	4 (10.8)	1 (4.8)	3 (18.8)	0.296
Hemoglobin, g/dL	10.5 ± 1.1	10.2 ± 1.0	10.8 ± 1.2	0.146
Albumin, g/dL	3.8 ± 0.4	3.8 ± 0.5	3.9 ± 0.2	0.477
Creatinine, mg/dL	10.1 ± 2.3	10.4 ± 2.2	9.8 ± 2.4	0.477
Calcium, mg/dL	8.7 ± 0.7	8.8 ± 0.7	8.7 ± 0.7	0.628
Phosphate, mg/dL	4.9 ± 1.4	4.8 ± 1.5	5.0 ± 1.4	0.672
URR	75.0 ± 3.9	75.0 ± 4.0	75.1 ± 3.9	0.910
Kt/V	1.67 ± 0.20	1.67 ± 0.19	1.67 ± 0.2	0.963
BMI	21.4 ± 2.7	21.5 ± 3.0	21.3 ± 2.3	0.842
Skeletal muscle mass	24.4 ± 4.0	24.3 ± 4.2	24.5 ± 3.9	0.908
Hand grip (kg)	24.0 ± 8.4	26.1 ± 8.8	21.2 ± 7.1	0.076
Walking speed (m/s)	0.83 ± 0.22	0.83 ± 0.24	0.86 ± 0.20	0.478

Data are presented as number of patients (%) or mean ± standard variation

BMI, body mass index; HD, hemodialysis; URR, urea reduction ratio

### Effects of exercise on laboratory finding and physical performance

The results of laboratory findings and physical performance tests at baseline and three months follow-up exercise were compared using a paired t-test. In the exercise group, the change in serum creatinine levels (9.8 ± 2.4 vs. 9.8 ± 2.6 mg/dL, *p* = 0.852) was not significant. The albumin levels (3.9 ± 0.2 vs. 4.0 ± 0.2 g/dL, *p* = 0.024) significantly increased in the exercise group. The hand grip strength significantly decreased in the non-exercise group (26.1 ± 8.8 vs. 22.1 ± 8.4 kg, *p* = 0.001) and increased in the exercise group (21.2 ± 7.1 vs. 23.8 ± 7.2 kg, *p* < 0.001) after exercise for three months. The walking speed also improved significantly in the exercise group (0.86 ± 0.20 vs. 1.14 ± 0.23 m/s, *p* < 0.001) (Table 2).

**Table 2:** 3-months changes of laboratory outcomes and physical function for participants

		Control (n = 21)	exercise (n = 16)
Cr (mg/dL)	Baseline (g/dL)	10.4 ± 2.2	9.8 ± 2.4
	After 3months (g/dL)	9.8 ± 2.7	9.8 ± 2.6
	*P* by paired t-test	0.098	0.852
Albumin (g/dL)	Baseline (mg/dL)	3.8 ± 0.5	3.9 ± 0.2
	After 3 months (mg/dL)	4.0 ± 0.4	4.0 ± 0.2
	*P* by paired t-test	0.060	0.024
Grip strength (kg)	Baseline (mg/dL)	26.1 ± 8.8	21.2 ± 7.1
	After 3 months (mg/dL)	22.1 ± 8.4	23.8 ± 7.2
	*P* by paired t-test	0.001	< 0.001
Walking speed (m/s)	Baseline (mg/dL)	0.83 ± 0.24	0.86 ± 0.20
	After 3 months (mg/dL)	0.71 ± 0.19	1.14 ± 0.23
	*P* by paired t-test	0.002	< 0.001

Data are presented as mean ± standard variation

Cr, creatinine

### Protein functional annotation and enrichment analysis

**Fig. 2:**
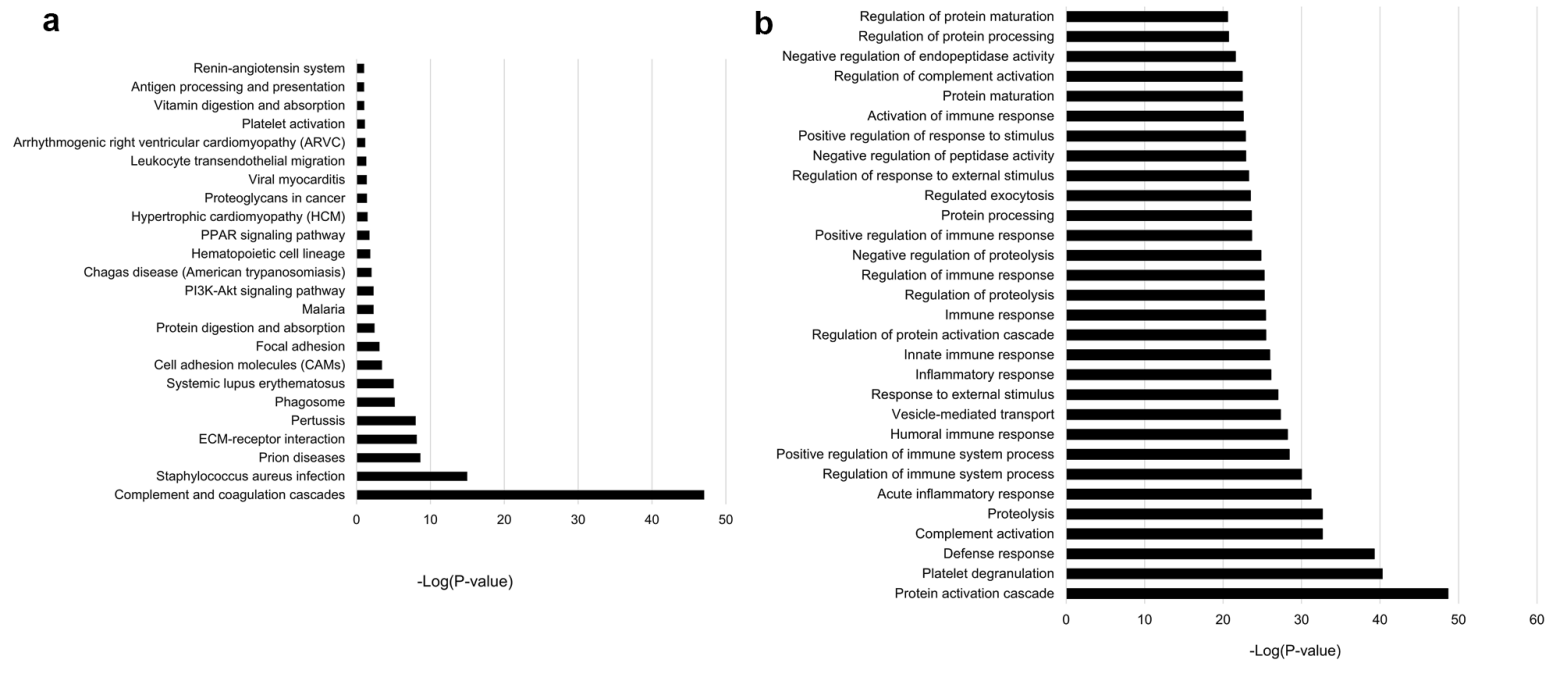
Plasma proteins that differentially expressed in before exercise and after 3 months exercise (**A**) Volcano plot shows the fold changes and associated *P* – values. (Dashed line indicates significance threshold, *P* = 0.05). (**B**) The concentration scale for 60 significantly differentially expressed proteins, which were converted from the quantitative data based on MS analysis

In the KEGG pathway analysis of DEPs, the significantly enriched proteins were those associated with processes, such as complement and coagulation cascades, extracellular matrix-receptor interactions, phagocytosis, cell adhesion, and protein digestion and absorption. The top 24 KEGG pathways are shown in Fig. 2A.

In the enrichment analysis for DEPs using GO pathway analysis (Fig. 2B), the 10 most prevalent pathways were protein activation, platelet deregulation, defense response, complement activation, proteolysis, acute inflammatory response, regulation of immune system process, positive regulation of immune system process, humoral immune response, and vesicle mediated transport. The proteolysis pathway contained *FCN3*, *ADAMDEC1*, *SERPINE2*, *PROS1*, *FETUB*, *ADAMTS13*, *PCSK9*, *MMP9*, *F7*, and *COL6A3* genes. The regulation of immune system process pathway contained *ITGB1*, *IGHM*, *ORM1*, *FCN3*, *ECM1*, *ACVR1B*, *IGF2*, *DPP4*, *C3*, and *VCAM1* genes. The positive regulation of immune system process pathway contained *IGHM*, *FCN3*, *PGLYRP2*, *TFRC*, *IGHV3-23ICAM1*, *ACVR1B*, *C3*, *VCAM1*, and *IGF1* genes. These findings suggest that protein expression associated with the immune response is affected by exercise in patients on dialysis.

**Fig. 3:**
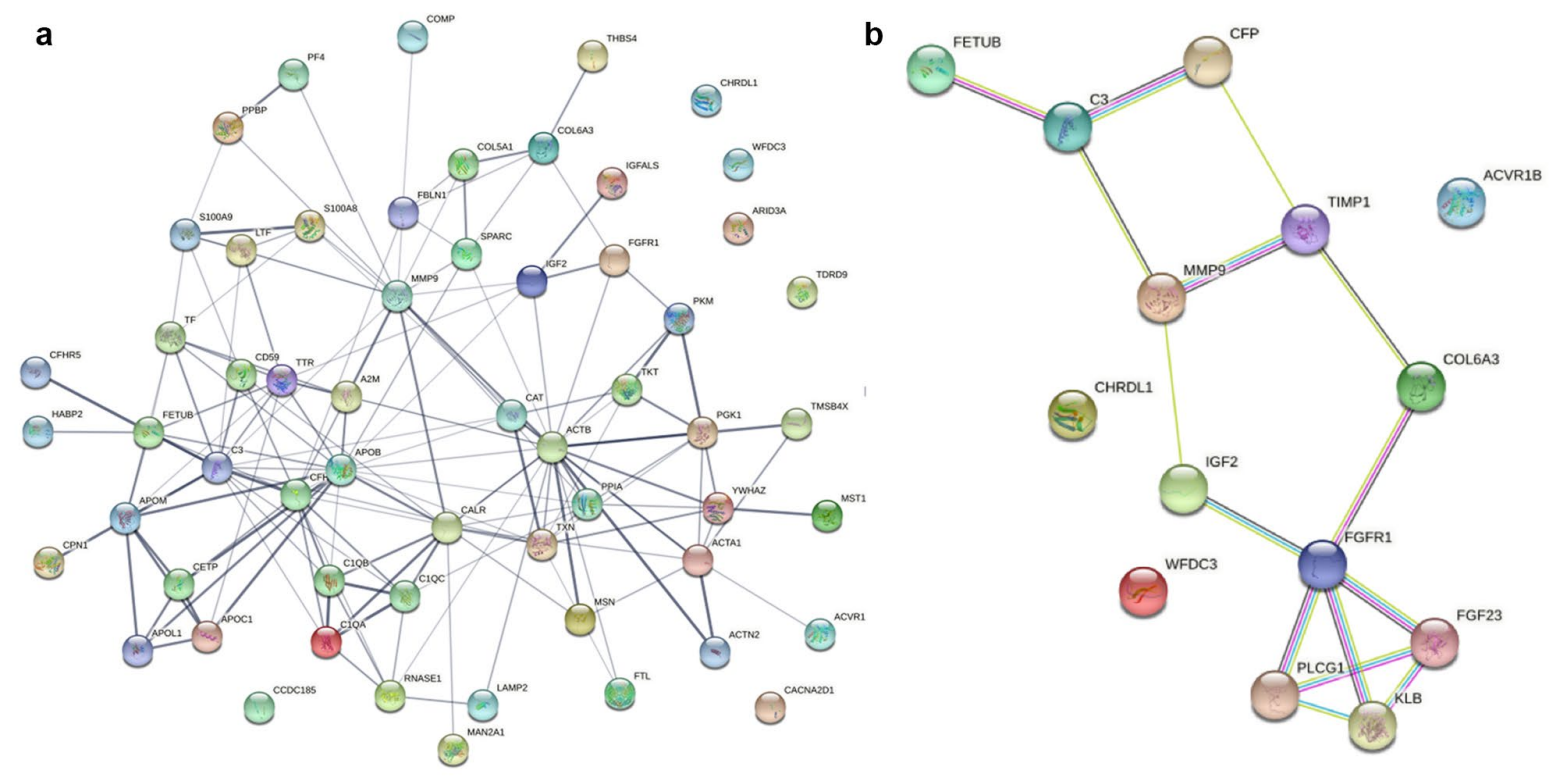
The KEGG pathway and GO analysis of significantly expressed proteins. The vertical axis represents the pathway category and the horizontal axis represents the enrichment score [− log(P-value)] of the pathway. Significantly enriched pathways (P < 0.05) are presented. The data were analyzed by DAVID bioinformatics tools. (**A**) The top 24 most functionally enriched KEGG pathways found in before exercise vs. after 3 months exercise. (**B**) The top 30 enriched GO terms found in before exercise vs. after 3 months exercise.

The PPI network map showed that these proteins were involved in various biological processes such as neutrophil aggregation, regulation of membrane attack complex activation, regulation of chondrocyte proliferation, very-low-density lipoprotein particle clearance, and leukocyte aggregation and molecular functions, such as Toll-like receptor 4 binding, antioxidant activity, integrin binding, glycosaminoglycan binding, and sulfur compound binding (Fig. 3).

### Proteomics-based protein identification and quantification

**Fig. 4:**
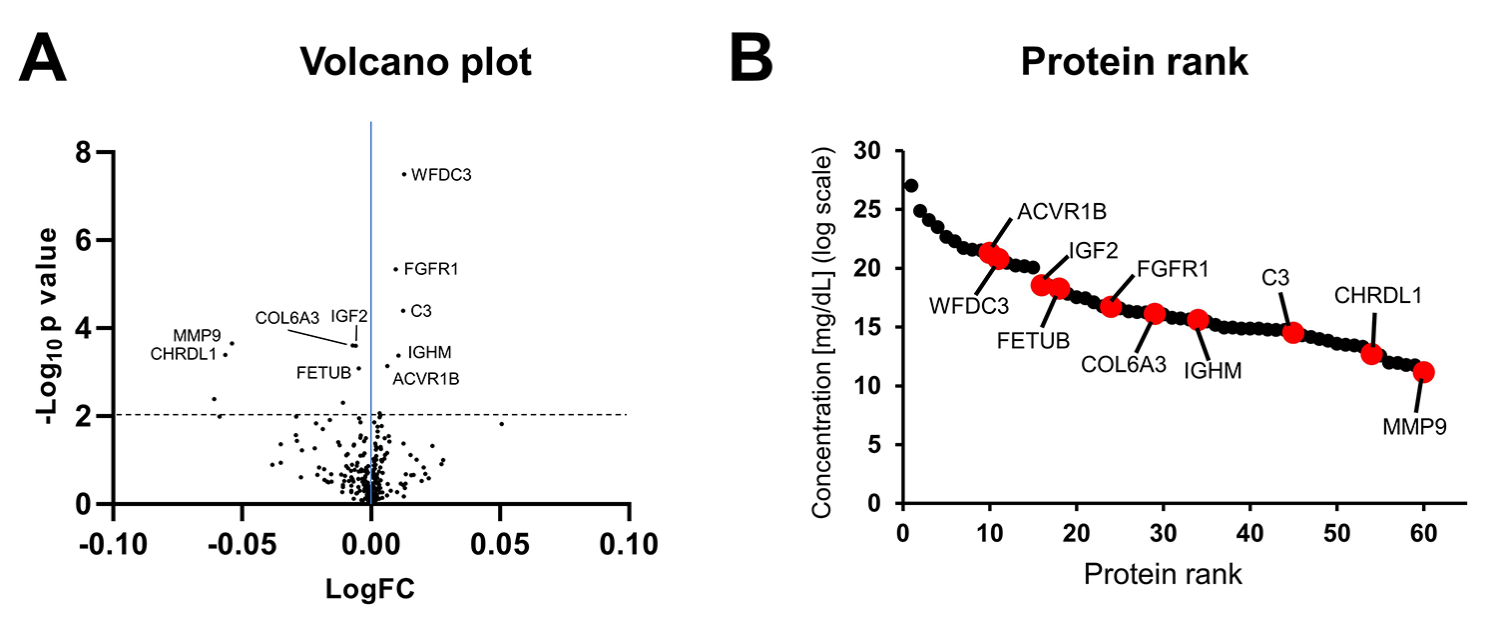
The protein–protein interaction network analysis was performed using Search Tool for the Retrieval of Interacting Genes/Proteins (STRING) software. The nodes reflect individual proteins enriched in the analysis, and the edges are the functional associations based on various online resources. (**A**) Protein-Protein interaction network of significantly expressed proteins that contains 58 nodes and 153 edges, with a PPI enrichment *P*-value < 1.0 x 10^− 16,^ line thickness indicates the strength of data support (The thickest edge indicates the highest confidence in protein-protein interaction). (**B**) Protein-Protein interaction network of top 10 proteins that contains 14 nodes and 15 edges, with a PPI enrichment P-value < 0.008, line color indicates the type of interaction evidence (light blue,known interactions from curated databases; purple, known interactions experimentally determined; green, gene neighborhood; red, gene fusions Blue, gene co-occurrence.)

To analyze the protein expression in the 16 patients before and after three months of exercise, DIA was performed by analyzing the samples in a randomized order. Finally, 433 proteins (200 upregulated and 233 downregulated, Fig. 4A) were qualified per sample; the peptides associated only with protein accessions with no gene association were excluded from the analysis (Table S1, S2). The profile plot shows that 433 proteins were quantified with high reproducibility across all LC-DIA-MS runs (Supplementary Fig. 1).

The plasma proteome profiles before and after exercise were compared, to determine significant differences in plasma protein expression, reflecting a significant effect of exercise in these patients. Paired t-test was performed to identify significantly DEPs between two intervals of three months of exercise. We found 60 significantly expressed proteins with a *p*-value < 0.05 (Fig. 4B, Table S3). Among these proteins, the top 10 proteins (five increased and five decreased) that were the most significantly different between before and after exercise were: (WFDC3 [*P* = 3.0 × 10^− 7^], FGFR1 [*P* = 4.8 × 10^− 6^], C3 [*P* = 4.2 × 10^− 5^], MMP-9 [*P* = 2.3 × 10^− 4^], COL6A3 [*P* = 2.6 × 10^− 4^], IGF2 [*P* = 2.5 × 10^− 4^], CHRDL1 [*P* = 4.2 × 10^− 4^], IGHM [*P* = 4.4 × 10^− 4^], ACVR1B [*P* = 7.6 × 10^− 4^], and FETUB [*P* = 8.6 × 10^− 4^].

### Validation of proteomics data for selected proteins by ELISA

Among the top 10 proteins that were significantly expressed and significantly enriched after exercise, MMP-9, ACVR1B, and FETUB were selected for the validation of proteomic data obtained using ELISA. The log_2_ fold changes (after three months of exercise vs. before exercise) for MMP-9, ACVR1B, and FETUB were found to be − 1.468583912, 0.311879039, and − 0.193814099, respectively, with all *p* values < 0.05 (Table S2).

**Fig. 5:**
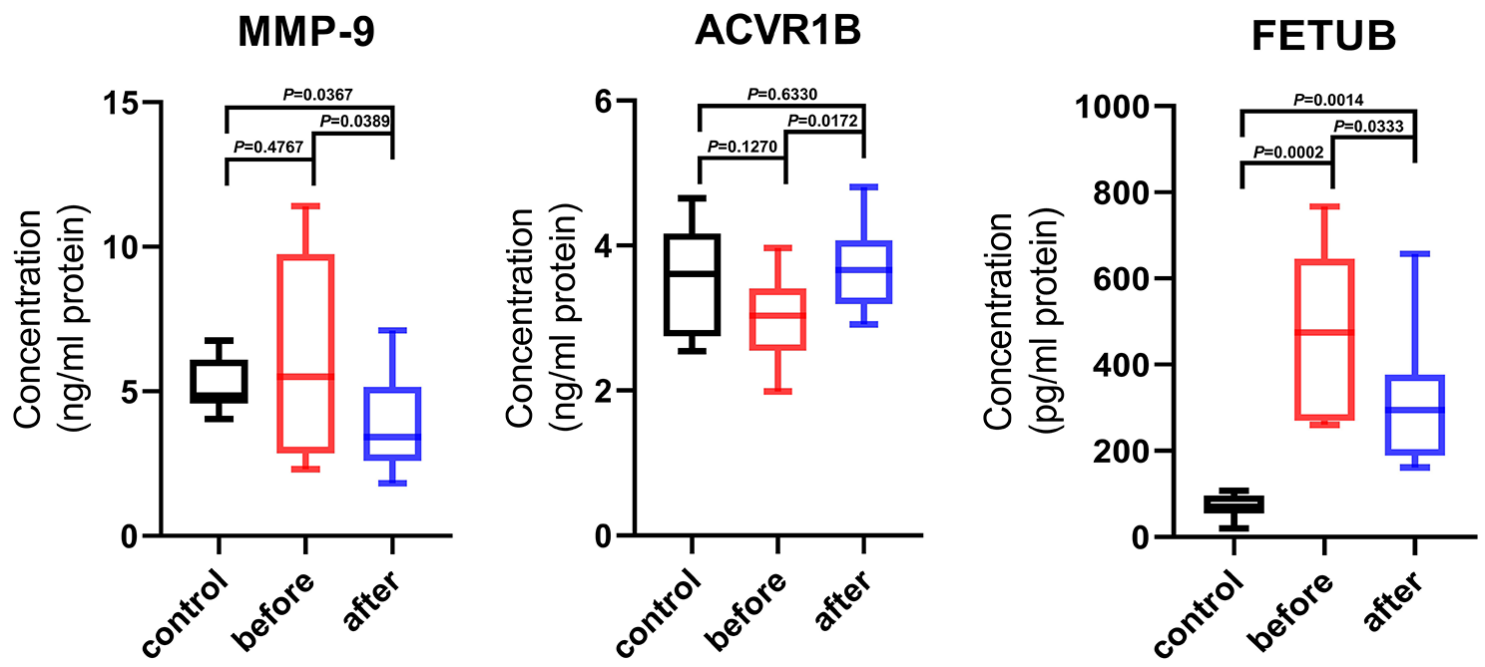
Validation of proteomics results with ELISA. Age-matched healthy control, before exercise, and after 3 months exercise. *P < control vs. before exercise; **P < control vs. before exercise; ***P < before exercise vs. after exercise. Control, Age-matched healthy control; before, before exercise in dialysis patients; after, after 3 months exercise in dialysis patients

The baseline levels of MMP-9 and FETUB were higher in patients with CKD on dialysis than those in the healthy age-matched control group, and the expression level significantly decreased after exercise. In contrast, the level of ACVR1B was lower in patients on dialysis than that in healthy controls, and the level significantly increased from the baseline after three months of exercise (Fig. 5).

## Discussion

The present study investigated the effects of exercise training on protein expression in patients on hemodialysis by comparing the altered protein expression levels before and after the exercise intervention. The significantly enriched proteins were involved in the immune response on protein functional analysis. Some main proteins, which demonstrated significant changes, were also validated through ELISA. In addition, the protein expression levels of patients on dialysis were compared with those of a healthy age-matched control group, confirming the effect of exercise on improving the levels of these proteins.

CKD is an independent risk factor for various adverse health issues, particularly CVDs. The age-adjusted mortality of CVD is much higher than the mortality of the general population, ranging approximately 15–30 times [14, 15]. Several mechanisms of this phenomenon have been suggested [16]. Among the factors involved, physical inactivity is a modifiable risk factor for morbidity and mortality. It is also a health-related quality [17].

Previous studies have reported a relationship between poor physical activity and high mortality in patients with CKD. Several trials have shown the effect of exercise on improving physical function and outcomes in patients with CKD. The largest randomized clinical trial conducted by Rossi et al. showed that 12-week/24-session renal rehabilitation exercise intervention improved the quality of life and physical function [18]. Several randomized trials on patients with ESRD receiving dialysis reported improvements in physical performance measures [19]. The present study also showed that exercise intervention for 12 weeks significantly improved physical function, measured by hand grip strength and 4 m walking speed, in patients with ESRD on maintenance dialysis treatment.

Although studies investigating the effects of exercise on improving cardiovascular risk in patients with CKD are rare, some studies have reported beneficial cardiac outcomes observed in exercise interventions in this population [20–23]. In addition, exercise remarkably improves traditional cardiovascular risk factors such as glucose control, blood pressure, lipid profile, and visceral fat in patients with CKD [24–26].

Recent studies have suggested that the immune system plays an important role in cardiac function and composition [27]. The researchers explored the role of the immune system in various heart diseases, including coronary artery disease, heart failure, and arrhythmia, which are the main manifestations of CVD in patients with CKD. Ischemic injury caused by the rupture and obstruction of the coronary arterial walls mobilizes numerous innate and adaptive immune cells. The inflammatory cytokines produced by these cells induce immune reactions, recruiting neutrophils and monocytes to participate in the active inflammatory cascade.

Furthermore, previous studies have suggested that regular exercise could reduce pro-inflammatory cytokine secretion and even modulate the immune system, eventually reducing the risk of CVD and cancer [28] [29]. The current study performed protein functional annotation and enrichment analysis. Our findings also demonstrated that exercise in patients on dialysis would have affected proteins associated with the immune response. Further, validated proteins reported to be related to the immune response in previous studies also showed significant changes after exercise.

MMP-9 is secreted by different immune-related cells [30]. A previous cross-sectional study showed that MMP-9 levels were significantly higher in patients with acute coronary syndrome than in those with stable angina [31]. Marat et al. suggested that MMP-9 levels were positively associated with the necrotic core size of coronary atherosclerotic plaques [32]. In the present study, MMP-9 levels were higher in patients on dialysis than that in healthy controls. The levels decreased significantly after exercise, suggesting a favorable effect of exercise on lowering MMP-9 levels. This result is similar to that of a previous study by Filipovic et al., which showed that a 12-week exercise program decreases the activity of serum MMP-1 [33] in humans. Also, Shon et al. showed that treadmill exercise attenuates MMP-9 activity in preexisting atherosclerotic plaque in an animal model [34].

The serum level of fetuin-B, the protein encoded by the *FETUB* gene, is increased in patients with coronary artery disease [35]. Fetuin is a cysteine protease inhibitor involved in inflammation and a potential player in proteolytic networks controlling immune defense, inflammation, and fibrosis [36]. A previous study showed that the serum fetuin B expression level is higher in patients with acute myocardial infarction (AMI) than in those with stable angina. Fetuin-B has been suggested as a therapeutic target for patients at a high risk of AMI [37]. The present study also showed that FETUB levels are higher in patients with ESRD on dialysis and significantly decrease after exercise. Keihanian et al. previously demonstrated that 8 weeks of exercise in male patients with type 2 DM reduces fetuin-B levels [38], which has also been suggested as a causative agent of insulin resistance and the inflammation pathway, resulting in type 2 DM [39, 40].

Although studies investigating the effect of ACVR1B on CVD are scarce, it is known that ACVR1B belongs to the TGFT-b superfamily and contributes to the resolution of inflammation. In a gene analysis study, *ACVR1B* has been identified as a validated relevant gene of emphysema [41], a subtype of progressive airflow obstruction accompanied by chronic *inflammation*. ACVR1B has also been suggested as a growth factor in muscles [42], and a previous genetic mapping study has demonstrated a strong association between knee muscle strength and the ACVR1B genotype [43]. The present study showed that ACVR1B levels were lower in patients on dialysis than in healthy controls. However, the levels significantly increased after exercise, suggesting the beneficial effect of exercise on ACVR1B levels.

There are some limitations of the study that warrant discussion. First, the study population was small, and the duration of exercise intervention was relatively short. Further investigations involving a larger number of patients over a longer period of exercise intervention are needed to prove the immune response-related beneficial effects of exercise. Second, we could not analyze the protein expressions of the healthy age-matched control group. However, we strengthened the results by confirmation using ELISA validation with this control group. In addition, we could not investigate the direct correlation between improvement in physical function and the levels of expressed proteins. As no previous studies have investigated changes in protein levels after exercise using proteomic profiling, the present study, which evaluated the effect of exercise by analyzing molecular level changes using a high-confidence proteomic method, would be meaningful. We also used functional analysis to confirm the potential role of the significantly changed proteins and strengthened the results by confirmation using ELISA analysis.

In conclusion, exercise in dialysis-dependent patients with CKD could enhance physical activity, which is a modifiable factor associated with reducing cardiovascular risk and mortality in the study population. The proteomic profiling results of protein expression changes after exercise might imply that the immune response is associated with this change in these patients. To investigate the beneficial effects of exercise on improving the outcomes of patients with ESRD on dialysis, we need more studies with longer intervention periods and larger patient populations.

## Electronic supplementary material

Below is the link to the electronic supplementary material.

Supplementary Material 1

Supplementary Material 2

Supplementary Material 3

Supplementary Material 4

## Data Availability

The authors confirm that the data supporting the findings of this study are available within the article and its supplementary materials.

## List of Abbreviations

CKD: Chronic kidney disease
CVD: Cardiovascular disease
DDA: Data dependent acquisition
DEPs: Differentially expressed proteins
DIA: Data-independent acquisition
ELISA: Enzyme linked immunosorbent assay
ESRD: End-stage renal disease
GO: Gene ontology
HRM: Hyper reaction monitoring
Ig: Immunoglobulin
KEGG: Kyoto Encyclopedia of Genes and Genomes
LC-MS/MS: Liquid Chromatography with tandem mass spectrometry
MARS: Multiple affinity removal system
PPI: Protein-protein interactions
