# Supplementary material for: Proteomic profiling of protein expression changes after 3 months-exercise in ESRD patients on hemodialysis
Source: BMC Nephrol. 2023 Apr 21;24:102. doi: 10.1186/s12882-023-03146-w (PMC10122383; doi:10.1186/s12882-023-03146-w)
Supplement: Supplementary file 3 — Supplementary Material 3 [file 12882_2023_3146_MOESM3_ESM.docx]

Supplementary Figure 1.

Reproducibility of LC-DIA runs before normalization (A) and after normalization (B)
